# Activation of mTORC1 by LSECtin in macrophages directs intestinal repair in inflammatory bowel disease

**DOI:** 10.1038/s41419-020-03114-4

**Published:** 2020-10-26

**Authors:** Qian Li, Hanxing cheng, Yuanping Liu, Xiaowen Wang, Fuchu He, Li Tang

**Affiliations:** 1 Institute of Biomedical Sciences, Fudan University, 200032 Shanghai, China; 2 State Key Laboratory of Proteomics, Beijing Proteome Research Center, National Center for Protein Sciences (Beijing), Beijing Institute of Lifeomics, 102206 Beijing, China; 3 Department of Biochemistry and Molecular Biology, Anhui Medical University, 230032 Hefei, China

**Keywords:** TOR signalling, Cell death and immune response, Phagocytes

## Abstract

Damage to intestinal epithelial cells and the induction of cellular apoptosis are characteristics of inflammatory bowel disease. The C-type lectin receptor family member LSECtin promotes apoptotic cell clearance by macrophages and induces the production of anti-inflammatory/tissue growth factors, which direct intestinal repair in experimental colitis. However, the mechanisms by which the phagocytosis of apoptotic cells triggers the pro-repair function of macrophages remain largely undefined. Here, using immunoprecipitation in combination with mass spectrometry to identify LSECtin-interacting proteins, we found that LSECtin interacted with mTOR, exhibiting a role in activating mTORC1. Mechanistically, apoptotic cells enhance the interaction between LSECtin and mTOR, and increase the activation of mTORC1 induced by LSECtin in macrophages. Elevated mTORC1 signaling triggers macrophages to produce anti-inflammatory/tissue growth factors that contribute to the proliferation of epithelial cells and promote the reestablishment of tissue homeostasis. Collectively, our findings suggest that LSECtin-dependent apoptotic cell clearance by macrophages activates mTORC1, and thus contributes to intestinal regeneration and the remission of colitis.

## Introduction

Inflammatory bowel disease (IBD) is a chronic inflammatory condition of the gastrointestinal tract caused by impairment of the intestinal epithelial cell (IEC) layers^1–3^. Increasing evidence suggests that macrophages have protective functions in intestinal health and disease^4–6^. Macrophages engulf apoptotic cells that were produced by tissue damage. Then macrophages undergo a functional switch, produce anti-inflammatory/tissue growth factors that are essential for mucosal healing, and return to homeostasis^6–9^. Therefore, macrophages represent a potential therapeutic target in IBD^4^. However, the molecular mechanisms of macrophages confer protection during intestinal disease and how macrophages might be targeted therapeutically are not yet fully understood.

Myeloid C-type lectin receptors (CLRs) are particularly involved in the clearance of dying cells and cell debris^10^. These CLRs detect molecules released from dying cells or exposed by cell corpses and can integrate signaling that either suppresses or induces inflammation^11–13^. More is known about the signaling by receptors of necrotic cells and their functional consequences of uptake^14,15^. Apoptotic cells are generally considered to be immunologically silent in control of immunity and homeostasis^16–18^. The signaling pathways and functional outcomes of downstream CLRs, which sense apoptotic cells, are not understood. LSECtin (encoded by *Clec4g*), which belongs to the CLR superfamily, is a type II transmembrane protein that has been previously detected on liver-resident macrophages called Kupffer cells (KCs) and intestinal macrophages^12,19,20^. LSECtin acts as a coinhibitory molecule and limits T cell immunity to promote hepatitis B virus tolerance^20,21^. Similar to DC-SIGN and L-SIGN, LSECtin functions as an endocytic receptor and mediates the binding and internalization of relevant apoptotic cells and viruses^14,22^. Findings from our recent study revealed that LSECtin, which is located on intestinal macrophages, promotes macrophage phagocytosis of apoptotic cells, contributing to tissue homeostasis in colitis^12^. However, the molecular mechanisms by which LSECtin senses apoptotic cells and restores tissue homeostasis in macrophages have not been fully elucidated.

Mammalian target of rapamycin (mTOR) is an evolutionarily conserved serine/threonine kinase^23,24^ that senses and integrates a myriad of stimuli and plays a critical role in many biological processes, such as cell growth, proliferation, survival, and immune cell activation^25,26^. Upstream regulators, such as growth factors and amino acids, activate mTORC1, enhance protein synthesis through mTORC1-mediated phosphorylation of ribosomal protein S6 kinase (S6K) and eukaryotic translation initiation factor 4E-binding protein 1 (4EBP1), which are the best described downstream effectors, and induce cell-cycle progression and growth^24,27,28^. mTOR is the major metabolic regulator that senses intracellular and extracellular signaling and controls macrophage metabolism and activation^29,30^. Recent studies at the cellular level have revealed that mTOR can exert both inflammatory and anti-inflammatory effects, depending on the physiological context and cellular subsets^31–35^. The mTORC1 signaling pathway in parenchymal cells has been widely studied^36–38^, but functional studies of mTORC1 in nonparenchymal cells are relatively scarce and highly controversial, especially those related to mTORC1’s function in myeloid cells in IBD, which remain largely unknown.

Here, we identified mTOR as an interacting partner of LSECtin that is also activated by LSECtin. Furthermore, we found that mTOR interacts with LSECtin in macrophages in an apoptotic cell-regulated fashion, and that apoptotic cell-mediated activation of mTORC1 induces macrophages to produce anti-inflammatory/tissue growth factors and thus to contribute to tissue homeostasis. In addition, we used a DSS-induced colitis model to explore the protective role of apoptotic cell-LSECtin-mTORC1 signaling in intestinal injury. Thus, we concluded that LSECtin-expressing macrophages activated the mTORC1 signaling pathway after engulfing apoptotic cells, which promoted the proliferation of epithelial cells in a DSS-induced colitis model.

## Results

### Identification of mTOR as an LSECtin-interacting protein

**Fig. 1:**
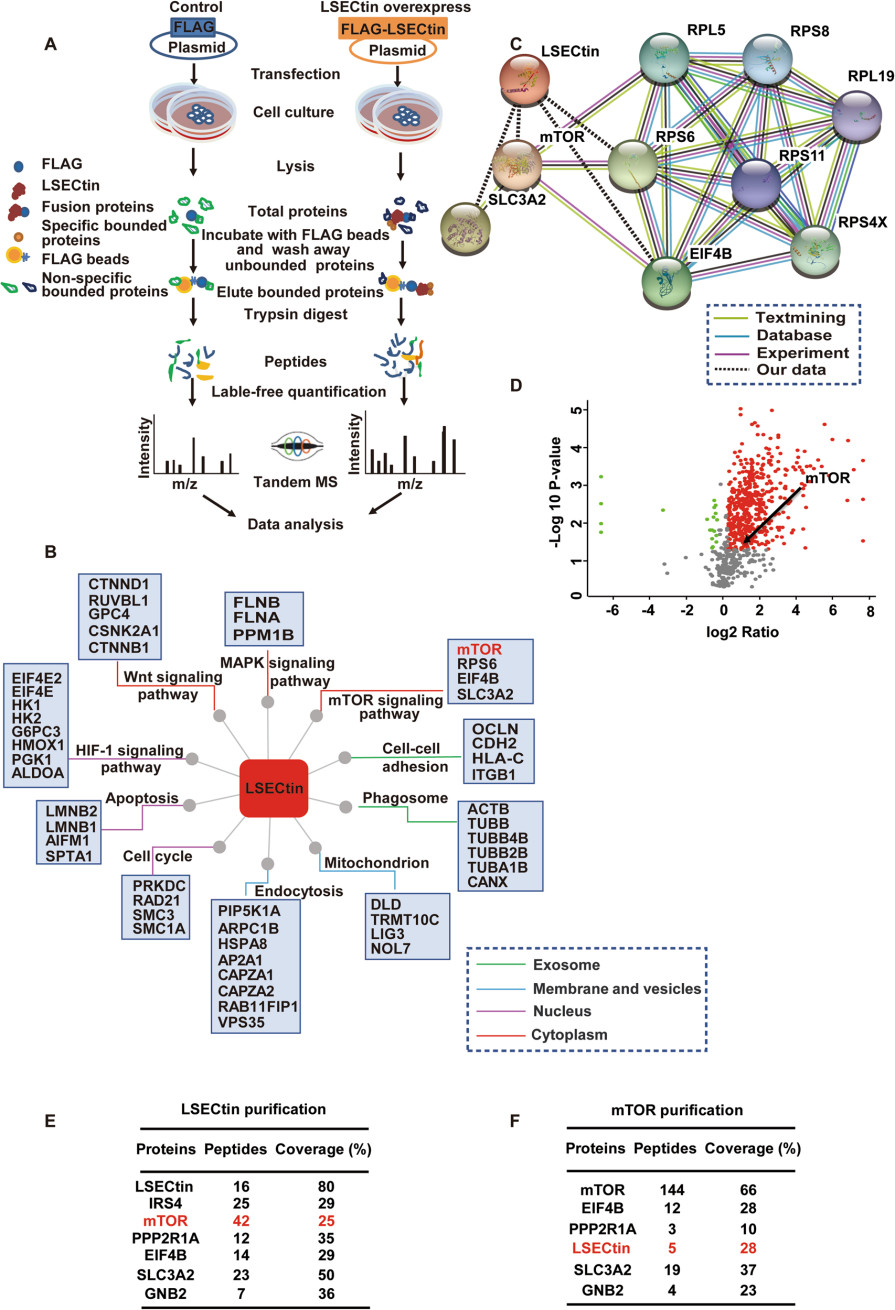
Identification of mTOR as an LSECtin-interacting protein. **a** Workflow of the immunoprecipitation procedure in combination with mass spectrometry used to identify hLSECtin-interacting proteins. **b** The hLSECtin-interacting proteins were grouped based on cellular component and biological process GO categories upon analysis. **c** PPI network of mTOR-related proteins and proteins captured by hLSECtin. **d** A volcano plot demonstrated the quantified proteins captured by hLSECtin. Proteins with a fold change >1.2 and a *P* value <0.05 (compared with purified control samples) were considered to be proteins specifically selected by hLSECtin. **e**, **f** The spectral counts of the hLSECtin-derived (**e**) and mTOR-derived (**f**) peptides detected by mass spectrometry in immunoprecipitants prepared from 293T and THP-1 cells overexpressing the indicated FLAG-tagged proteins. All experiments were repeated three times.

Our previous studies revealed that LSECtin, which is located in macrophages, facilitated macrophage phagocytosis of apoptotic cells to promote the proliferation of epithelial cells and ameliorate colitis^12^. To explore how LSECtin regulates macrophage-directed intestinal regeneration in experimental colitis, we sought to identify LSECtin-interacting proteins. We used immunoprecipitation in combination with mass spectrometry (IP-MS) to screen for LSECtin-interacting proteins^39,40^. The workflow of the IP-MS procedure used in this study is shown in Fig. 1a. FLAG-human LSECtin (hLSECtin) or an empty vector was overexpressed in human embryonic kidney 293T (HEK293T) cells, coimmunoprecipitated from whole-cell lysates (Fig. S1A), and separated by SDS-PAGE to reduce sample complexity (Fig. S1B) prior to the LC-MS/MS analysis. From the mass spectrometric analyses of anti-FLAG immunoprecipitants prepared from 293T cells overexpressing FLAG-tagged hLSECtin, we identified 1328, 1327, and 1312 proteins in three replicates (Fig. S1C). In addition, the Pearson correlation coefficient was calculated to determine the validity of the results. High reproducibility was observed in the abundance of total protein in three replicates, with a Pearson *r* value >0.95 (ranging from 0.99 to 1) (Fig. S1D). We quantified more than 1310 LSECtin-interacting proteins in triplicate screens, and based on analyses using the Gene Ontology (GO) database “Biological Process” domain, we found that these proteins are mainly involved in the mTOR signaling pathway, apoptosis, endocytosis, phagosomes, and other pathways (Fig. 1b). We focused on mTOR, which plays a critical role in many biological processes, such as immune cell activation^41^. A protein-protein interaction (PPI) network (STRING) analysis showed that four of the LSECtin-interacting proteins are involved in the mTOR signaling pathway (Fig. 1c). We consistently found mTOR protein among the candidate proteins (Fig. 1d, e).

In addition, to confirm the interaction between mTOR and LSECtin, we also performed reverse IP-MS experiments to screen for mTOR-interacting proteins. FLAG-mTOR was overexpressed in THP-1 cells (as shown in Fig. S1E, we observed that hLSECtin was expressed in THP-1 cells), and IP-MS experiments were performed as described above. The FLAG coimmunoprecipitation (co-IP) effect was observed (Fig. S1F). From the results of the mass spectrometric analyses of anti-FLAG immunoprecipitants prepared from THP-1 cells overexpressing FLAG-tagged mTOR, we consistently detected hLSECtin protein among the candidate proteins (Fig. 1f). In summary, we identified mTOR as a potential interacting partner of LSECtin.

### Validation of mTOR as an LSECtin-interacting protein

**Fig. 2:**
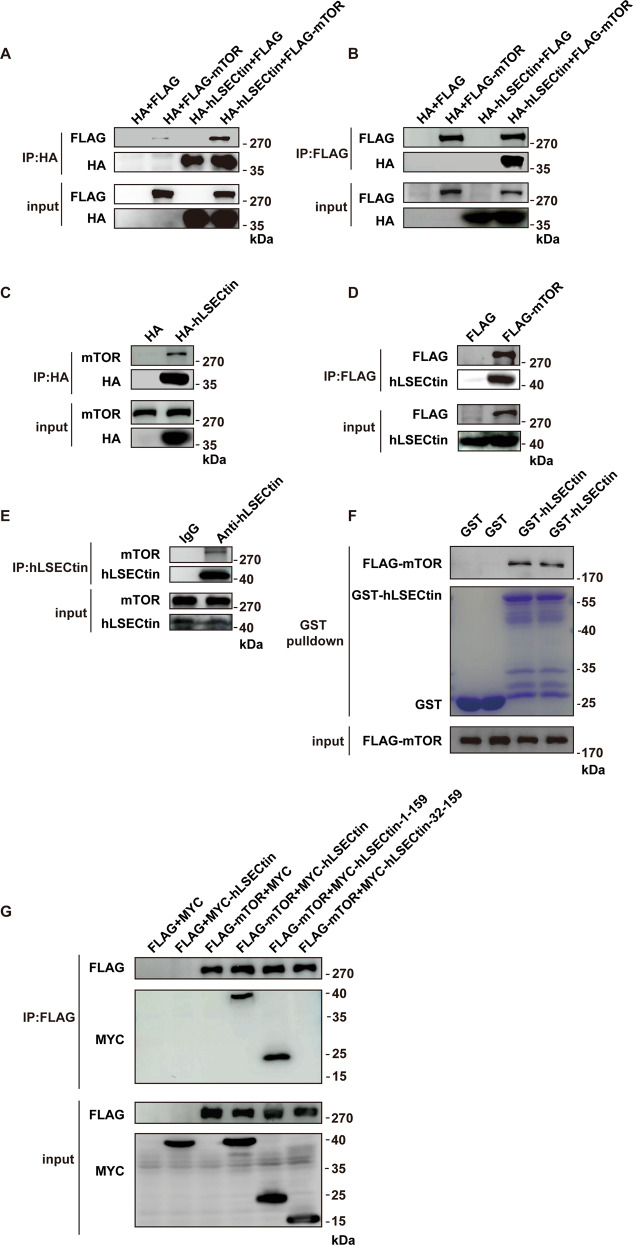
Validation of mTOR as an LSECtin-interacting protein. Co-IP assays were performed to confirm the interaction between LSECtin and mTOR. **a** 293T cells were transfected with HA-hLSECtin together with FLAG-mTOR or an empty vector. Lysates coimmunoprecipitated with anti-HA affinity gel were analyzed by immunoblotting with anti-FLAG or anti-HA. **b** 293T cells were transfected with HA-hLSECtin together with FLAG-mTOR or an empty vector. Lysates coimmunoprecipitated with anti-FLAG were analyzed by immunoblotting with anti-HA or anti-FLAG. **c** Overexpressed hLSECtin coimmunoprecipitated with endogenous mTOR components. Coimmunoprecipitates were prepared from 293T cells overexpressing the indicated HA-tagged proteins and analyzed along with cell lysates by immunoblotting for detection of the indicated proteins. **d** Overexpressed mTOR coimmunoprecipitated endogenous hLSECtin components. Coimmunoprecipitates were prepared from THP-1 cells overexpressing the indicated FLAG-tagged proteins and were analyzed along with cell lysates by immunoblotting for detection of the indicated proteins. **e** Cell lysates from THP-1 cells were coimmunoprecipitated with rabbit IgG or anti-hLSECtin antibody and immunoblotted with anti-LSECtin or anti-mTOR antibody. **f** GST-hLSECtin were purified from *E. coli* and FLAG-mTOR were purified from 293T cells, and then the in vitro GST pull-down assay were performed, as described in the “Materials and methods” section. **g** Key residues in the N-terminal domain of hLSECtin required for it to interact with mTOR were identified. 293T cells were transfected with MYC-hLSECtin or the indicated MYC-hLSECtin mutants together with FLAG-mTOR or an empty vector. Lysates coimmunoprecipitated with anti-FLAG were analyzed by immunoblotting with anti-FLAG or anti-MYC. All experiments were repeated three times.

As the initial step in verifying our mass spectrometric identification of mTOR as an LSECtin-interacting protein, we coexpressed HA-hLSECtin with FLAG-mTOR in 293T cells and found that hLSECtin coimmunoprecipitated with mTOR (Fig. 2a). We also found that mTOR coimmunoprecipitated with hLSECtin (Fig. 2b). Furthermore, when HA-hLSECtin was expressed in the 293T cells, HA-hLSECtin coimmunoprecipitated with endogenous mTOR (Fig. 2c). Correspondingly, FLAG-mTOR coimmunoprecipitated with endogenous hLSECtin from the THP-1 cells overexpressing FLAG-mTOR (Fig. 2d). In addition, the endogenous hLSECtin in the THP-1 cells coimmunoprecipitated with mTOR (Fig. 2e). Furthermore, the interaction between hLSECtin and mTOR was confirmed by in vitro glutathione *S*-transferase (GST) pull-down assay (Fig. 2f). LSECtin is a type II transmembrane protein. To identify the region in LSECtin that interacts with mTOR, we designed a truncated mutant of hLSECtin^42^ (Fig. S2A). We showed that the interaction of hLSECtin and mTOR was mediated through the N-terminal region of LSECtin as a deficiency of the first 31 amino acids in hLSECtin (amino acids 32-159) abolished its association with mTOR (Fig. 2g). Similarly, we also coimmunoprecipitated endogenous mTOR in mouse LSECtin (mLSECtin)-expressing mouse macrophage RAW264.7 cells (Fig. S2B). In addition, colocalization of LSECtin and mTOR was shown in mLSECtin-expression RAW264.7 cells after they engulfed apoptotic cells (Fig. S2C).

As expected from the mass spectrometry results, these findings validated mTOR as an LSECtin-interacting protein. More importantly, we found that the cytosolic N-terminal region of LSECtin was required for the LSECtin-mTOR interaction.

### LSECtin facilitates the activation of mTORC1 signaling in vitro and in vivo

**Fig. 3:**
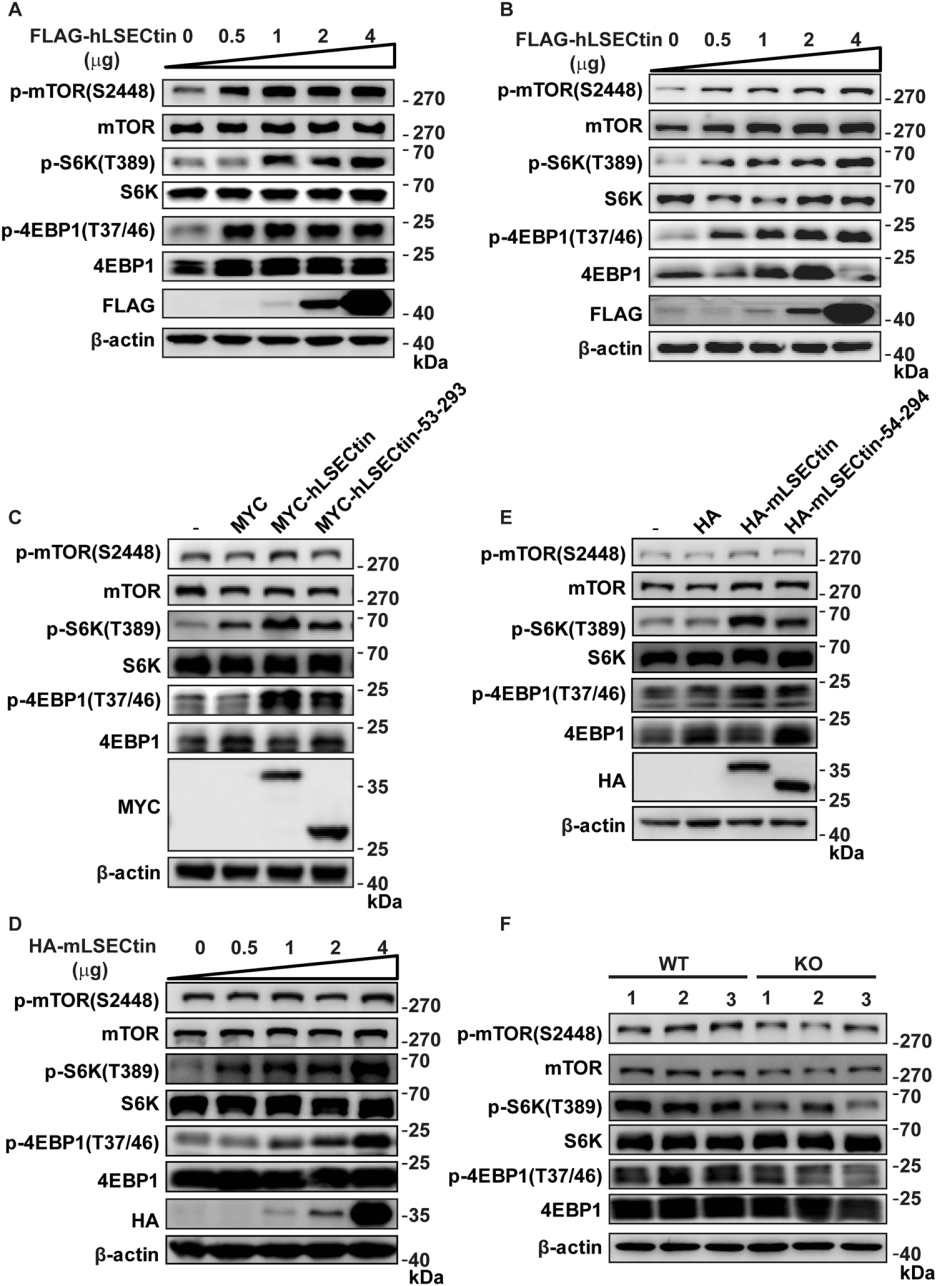
LSECtin facilitates the activation of mTORC1 signaling in vitro and in vivo. The phosphorylation status of mTOR, S6K, and 4EBP1 was examined by western blot analyses in the following cells: **a**, **b** 293T cells (**a**) and THP-1 cells (**b**) overexpressing the indicated FLAG-hLSECtin plasmid concentration for 48 h; **c** 293T cells overexpressing the indicated MYC-hLSECtin mutants for 48 h; **d**, **e** RAW264.7 cells overexpressing the indicated HA-mLSECtin plasmid concentration (**d**) and HA-mLSECtin mutants (**e**) for 48 h; **f** Colonic LPMCs isolated from LSECtin-WT and LSECtin-KO mice. All experiments were repeated three times.

Given the interaction between LSECtin and mTOR, what role does LSECtin play in the mTOR pathway? To investigate the function of LSECtin in the mTOR pathway, we overexpressed FLAG-hLSECtin at different levels in 293T cells. We found that hLSECtin could activate mTORC1 as detected in the phosphorylation of its downstream and effector proteins, including p-S6K^T389^and p-4EBP1^T37/46^. In addition, LSECtin facilitated mTOR activation in a dose-dependent manner (Fig. 3a). We also observed similar results in THP-1 cells (Fig. 3b). Furthermore, truncated hLSECtin mutants were overexpressed in 293T cells to detect the activation of mTORC1. We found that deficiency of the cytosolic N-terminal region of hLSECtin (amino acids 53–293) reduced the activation of mTORC1. (Fig. 3c and Fig. S3A). Besides, we found that the full length of hLSECtin and its intracellular fragments (amino acids 1–159) could be located on the cell membrane, when the full length or intracellular fragments of hLSECtin were overexpressed in 293T and THP-1 cells (Figs. S3B and S3C).

To verify that mLSECtin had the same effects as hLSECtin on mTORC1 activation, we overexpressed HA-mLSECtin at different levels in RAW264.7 cells. Similar to hLSECtin, mLSECtin activated mTORC1, with the degree of activation being proportional to the amount of mLSECtin expressed (Fig. 3d). We also found that deficiency of the cytosolic N-terminal region of mLSECtin (amino acids 54–294) reduced the activation of mTORC1 (Fig. 3e and Fig. S3D).

We isolated lamina propria mononuclear cells (LPMCs) from the colons of LSECtin wild-type (WT) and knock-out (KO) mice and detected the activation levels of mTORC1. Given that LSECtin is highly expressed in colonic macrophages^12^, we used LPMCs to represent colonic macrophages roughly. As expected, LSECtin was detected in the LSECtin-WT mice but not in the LSECtin-KO mice (Fig. S3E). We also found that mice depleted of LSECtin displayed attenuated mTORC1 activity in their LPMCs in vivo (Fig. 3f). In summary, these results indicated that LSECtin facilitates the activation of mTORC1 signaling in vitro and in vivo.

### Apoptotic cells enhance the LSECtin-mTOR interaction and mTORC1 activation

**Fig. 4:**
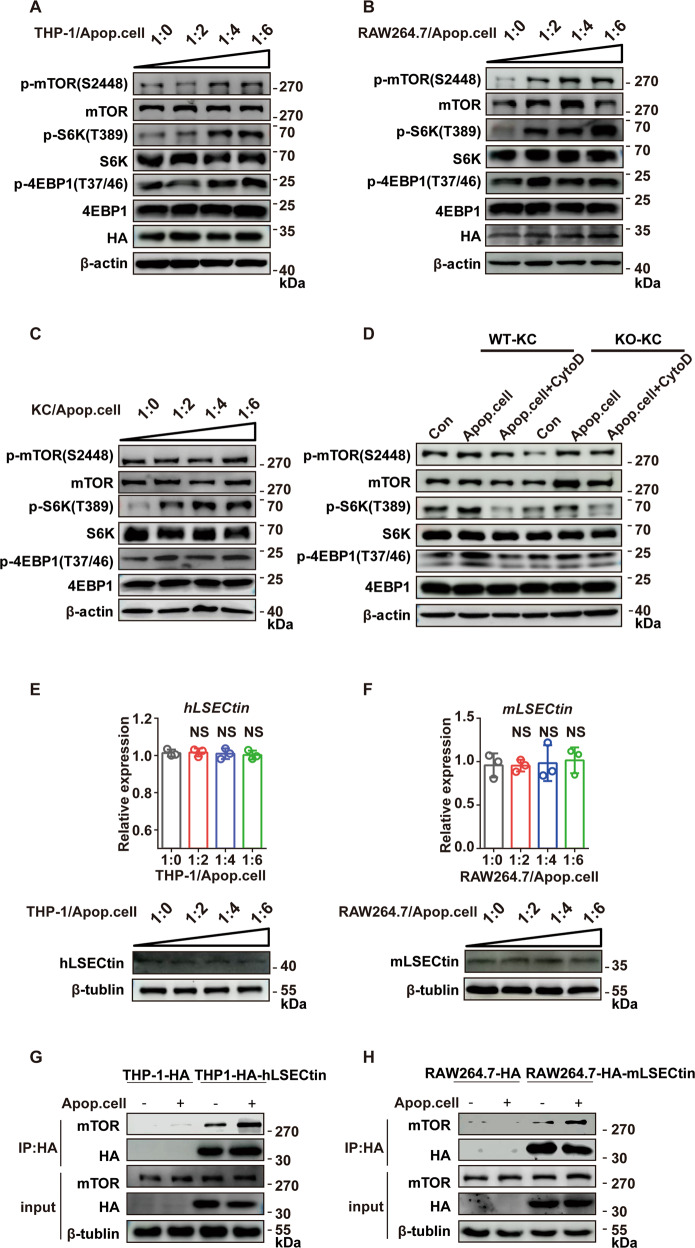
Apoptotic cells enhance the LSECtin-mTOR interaction and mTORC1 activation. **a**-**d** Phosphorylation status of mTOR, S6K, and 4EBP1 was determined by western blot analyses in the following cells: PMA-stimulated HA-hLSECtin-expressing THP-1 cells were treated with apoptotic HCT116 cells at the indicated ratios (**a**), HA-mLSECtin-expressing RAW264.7 cells were treated with apoptotic thymocytes at the indicated ratios (**b**), liver KCs were treated with apoptotic thymocytes cells at the indicated ratios (**c**), and liver KCs were either untreated or treated with 6× apoptotic thymocyte cells or CytoD (**d**). All experiments were repeated three times. **e**, **f** LSECtin expression was determined by quantitative RT-PCR (qRT-PCR) and western blot analyses in the following cells: PMA-stimulated THP-1 cells were treated with apoptotic HCT116 cells at the indicated ratios (**e**), and RAW264.7 cells were treated with apoptotic thymocytes at the indicated ratios (**f**). *n* = 3. Gene expression in THP-1 and RAW264.7 cells treated or untreated with apoptotic cells for 12 h (qRT-PCR) and 20 h (western blot) were measured by qRT-PCR or western blot analyses. All genes were normalized to *Gapdh*. Data are presented as the means ± SEM, calculated by unpaired, two-tailed Student’s *t*-tests. NS: no significant difference. Experiment was repeated three times. **g** PMA-stimulated HA-hLSECtin-expressing THP-1 cells after engulfing apoptotic HCT116 cells were lysed. Lysates coimmunoprecipitated with anti-HA affinity gel were analyzed by immunoblotting with the indicated antibody. **h** HA-mLSECtin-expressing RAW264.7 cells after engulfing apoptotic thymocytes were lysed. Lysates coimmunoprecipitated with anti-HA affinity gel were analyzed by immunoblotting with the indicated antibody. All experiments were repeated three times.

The number of apoptotic enterocytes has been reported to be increased in the colons of IBD patients^43^. The clearance of apoptotic cells is critical for maintaining tissue homeostasis. LSECtin expressed on macrophages facilitates the phagocytosis of apoptotic cells by macrophage and promotes the proliferation of epithelial cells in an engulfment-dependent manner. This engulfment progress by macrophages through LSECtin is critical for intestinal healing^12^. Whether apoptotic cells could activate mTORC1? THP-1 monocytic leukemia cells can be differentiated into macrophages using phorbol-12-myristate-13-acetate (PMA), as a reliable in vitro model^44,45^. As we predicted, apoptotic cells increased mTORC1 activation in PMA-stimulated hLSECtin-expressing THP-1 cells and in mLSECtin-expressing RAW264.7 cells, with the degree of activation being proportional to the number of apoptotic cells (Fig. 4a, b). We also found similar results in mouse liver KCs (Fig. 4c), and mTORC1 activation was attenuated when KC phagocytosis was suppressed by cytochalasin D (CytoD) (Fig. 4d). Furthermore, apoptotic cells could increase mTORC1 activation (reflected in the upregulation of its target genes p-S6K^T389^ and p-4EBP1^T37/46^) in LSECtin-WT KCs compared with LSECtin-KO KCs. How do apoptotic cells increase LSECtin-activated mTORC1 activity? Apoptotic cells could not significantly change the LSECtin expression in THP-1 cells and Raw264.7 cells (Fig. 4e, f). Besides, LSECtin expression levels on colonic lamina propria macrophages were not significantly altered during colitis progression^12^. Do apoptotic cells increase the interaction between LSECtin and mTOR to promote the activity of LSECtin-activated mTORC1? We found that in the presence of apoptotic cells, LSECtin coimmunoprecipitated with more endogenous mTOR in PMA-stimulated hLSECtin-expressing THP-1 cells and in mLSECtin-expressing RAW264.7 cells after engulfing apoptotic cells compared to controls (Fig. 4g, h).

In summary, we found that apoptotic cells enhance the interaction between LSECtin and mTOR, and promote the activation of mTORC1 by LSECtin.

### Elevated mTORC1 signaling promotes the pro-repair function of macrophages after phagocytosis of apoptotic cells

**Fig. 5:**
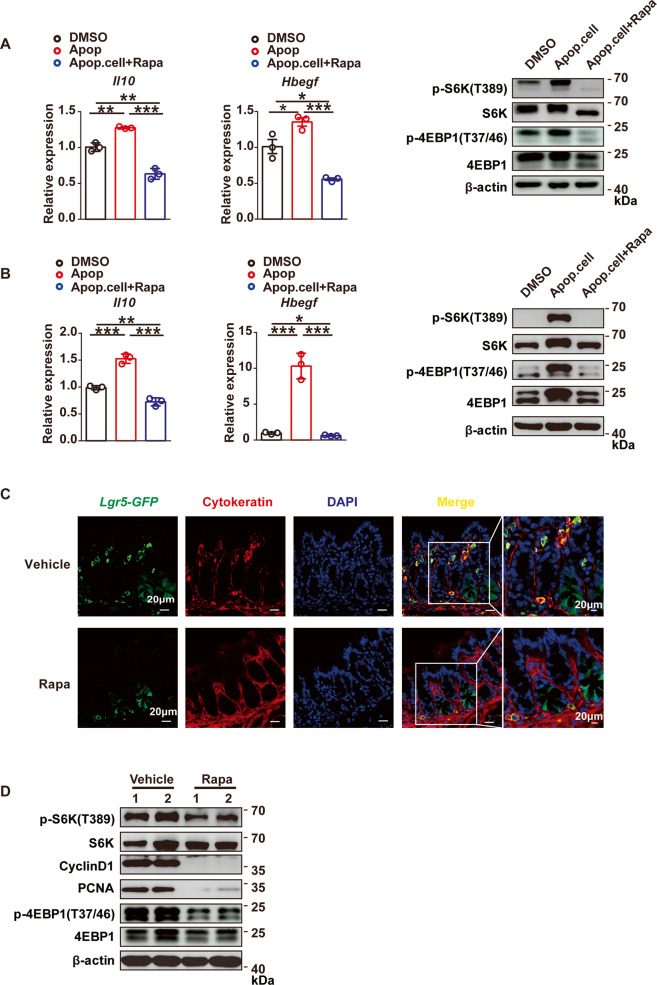
Elevated mTORC1 signaling promotes the pro-repair function of macrophages after phagocytosis of apoptotic cells. **a**, **b** Quantitative mRNA expression of *Il10* and *Hbegf* in WT KCs (**a**) and WT BMDMs (**b**) treated with apoptotic thymocytes with or without rapamycin for 4 h, the phosphorylation status of S6K and 4EBP1 was examined by western blot analyses. All genes were examined by qRT-PCR and normalized to *Gapdh*. *n* = 3. Experiment was repeated three times. Data were presented as the means ± SEM, calculated by unpaired, two-tailed Student’s *t*-tests; *P < 0.05; **P < 0.01; and ***P < 0.001. **c** Immunofluorescence staining for cytokeratin^+^ (red) cells, *Lgr5*^+^ cells (green), and nuclei in colon tissues from DSS-challenged WT mice treated or untreated with rapamycin (scale bar: 20 μm). **d** The phosphorylation status of S6K and 4EBP1, and the cyclin D1 and PCNA were examined by western blot analyses of colons from DSS-challenged WT mice treated with vehicle or rapamycin. All experiments were repeated three times.

Elevated mTOR signaling causes microglia to adopt a noninflammatory reactive phenotype and to promote the proliferation of astrocytes^35^. Additionally, bone marrow-derived macrophages (BMDMs) that had phagocytosed apoptotic cells showed decreased production of interleukin-6 (IL-6) after lipopolysaccharide stimulation^46,47^. LSECtin promotes apoptotic cell clearance by macrophages and induces intestinal regeneration and maintenance of the mucosal barrier after injury^12^. The data described above show that apoptotic cells regulate the LSECtin-mTOR interaction and promote mTORC1 activation. Next, we investigated the involvement of apoptotic cell-mediated activation of mTORC1 in macrophages producing anti-inflammatory/tissue growth factors. The mTOR inhibitor rapamycin has been widely used to investigate mTORC1 function and signaling^48–50^. KCs that had phagocytosed apoptotic cells showed increased the expression of anti-inflammatory/tissue growth factors *Il10* and *Hbegf*; in contrast, the expression of these genes was reduced by rapamycin treatment (Fig. 5a). We generated BMDMs (Fig. S4A) and confirmed that when mTORC1 activity was inhibited, the production of pro-resolving factors was impaired in BMDMs (Fig. 5b). We next investigated whether apoptotic cell-mediated mTORC1 activation was required for epithelial cell proliferation in vivo. Leucine-rich repeat-containing G-protein-coupled receptor 5 (LGR5) is a stem cell marker in various organs/tissues, including the gut^51^. *Lgr5*-expressing corpus cells drive epithelial regeneration post-injury^52^. We investigated the rate of colon regeneration in DSS-induced colitis with rapamycin intraperitoneally administered to *Lgr5-GFP* mice. Schematic for DSS-induced colitis in mice is shown in Fig. S4B. Our data showed that 6 days after the DSS challenge, IECs proliferation (the number of *Lgr5-GFP*^+^ cells) was markedly reduced in the mice in which mTORC1 activity had been inhibited compared with the level in control mice (Fig. 5c). Consistent with the increased rate of regeneration, proliferating cell nuclear antigen (PCNA) and cell-cycle genes encoding the late G1 to G1/S phase protein cyclin D1 were expressed at higher levels in vehicle mouse colonic tissues compared with colonic tissues in which mTORC1 activity had been inhibited (Fig. 5d).

Collectively, these findings in combination with our previous study indicated that the apoptotic cell-mediated activation of mTORC1 was required for the expression of anti-inflammatory/tissue growth factors in macrophages and participated intestinal regeneration in DSS-induced colitis.

### LSECtin promotes mTORC1 activation in colon macrophages and ameliorates DSS-induced colitis

**Fig. 6:**
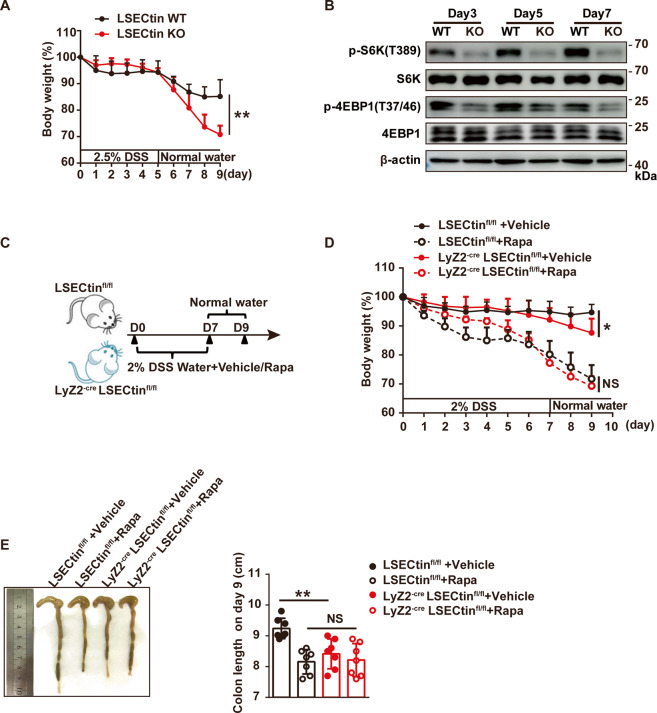
LSECtin promotes the activation of mTORC1 in colon macrophages and ameliorates DSS-induced colitis. **a** The body weight of DSS-challenged LSECtin-WT (*n* = 9) and LSECtin-KO (*n* = 10) mice were measured every day during colitis development. Data were presented as the means±SEM, followed by two-way analysis of variance (ANOVA); **P < 0.01. **b** The phosphorylation status of S6K and 4EBP1 was examined by western blot analyses of colonic LPMCs from LSECtin-WT and LSECtin-KO mice challenged with DSS for different durations (days). All experiments were repeated three times. **c** Schematic for DSS-induced colitis with or without rapamycin treatment. **d** LSECtin^fl/fl^ and Lyz2^-cre^ LSECtin^fl/fl^ mice treated with vehicle (LSECtin^fl/fl^, *n* = 7; Lyz2^-cre^LSECtin^fl/fl^, *n* = 8) or rapamycin (LSECtin^fl/fl^, *n* = 8; Lyz2^-cre^ LSECtin^fl/fl^, *n* = 8) were subjected to colitis induction with DSS. The change in body weight was measured every day, and the means were calculated. Data were presented as the means±SEM, followed by two-way analysis of variance (ANOVA); *P < 0.05; NS: no significant difference. **e** The lengths of the colons were measured on day 9. *n* = 6, 7, 7, 7. Data were calculated by unpaired, two-tailed Student’s *t*-tests; **P < 0.01; NS: no significant difference. Data were representative of three independent experiments with similar results (**a**, **d**, **e**).

mTORC1 signaling plays a crucial role in the regulation of various cellular processes in a number of pathological conditions^27,53^. mTORC1 plays a pivotal part in increasing the proliferation of intestinal epithelium and is required for intestinal regeneration against IBD^54^. However, the function of mTORC1 in myeloid cells during intestinal inflammation remains largely unknown. To explore the activation of mTORC1 in colonic macrophages during intestinal inflammation, DSS was added to mouse drinking water to induce colitis. LSECtin can ameliorate the colitis, the mean change in body weight was measured in Fig. 6a. Given that LSECtin is primarily expressed in colonic macrophages and no expression in IECs^12^, we used LPMCs to represent colonic macrophages roughly. We isolated LPMCs from the mouse colon at different points during IBD and detected mTORC1 activation. We found that mTORC1 activation gradually increased. In addition, LSECtin promoted mTORC1 activation as detected by p-S6K^T389^and p-4EBP1^T37/46^ in the macrophages of the mouse colon during IBD (Fig. 6b). Systemic injection of rapamycin into IBD mouse models inhibited intestinal regeneration, which was accompanied body weight loss^55^. mTORC1 activity inhibited by intraperitoneally injecting rapamycin every day during DSS administration in mice with macrophages (Mφs) lacking LSECtin (Lyz2^-Cre^ LSECtin^fl/fl^, LSECtin ΔMφs) and LSECtin-WT mice (LSECtin^fl/fl^) as shown in Fig. 6c. Administration of rapamycin and DSS caused similar effects on body weight loss and colon lengths in both LSECtin ΔMφ mice and LSECtin-WT mice (Fig. 6d, e), suggesting that LSECtin and mTOR may be involved in the same pathway and the DSS resistance of LSECtin-WT mice may mediate by mTORC1 signaling.

In summary, our results indicated that LSECtin-regulated mTORC1 signaling facilitated intestinal regeneration in DSS-induced colitis.

## Discussion

In this study, we identified mTOR as an LSECtin-interacting partner and found that mTORC1 can be activated by LSECtin. Mapping analysis showed that the central intracellular domain (ICD) of LSECtin was both sufficient and necessary for the interaction with mTOR, and LSECtin-activated mTORC1. Deletion of the ICD of LSECtin abolished the binding of LSECtin to mTOR and reduced the activation of mTORC1. Furthermore, apoptotic cells enhanced LSECtin-mediated mTORC1 activity by increasing the interaction between LSECtin and mTOR after they were engulfed by macrophages. Then, elevated mTORC1 signaling in these macrophages promoted the production of anti-inflammatory/tissue growth factors, facilitated the proliferation of IECs, and ameliorated DSS-induced colitis.

The mTOR pathway could be activated in diverse manners, including variations in the ATP:AMP ratio via AMP-activated protein kinase, insulin and amino acids, Rag GTPases, and Wnt activation of the glycogen-synthase kinase 3 (GSK3) pathway^23,26,56,57^. Immune system receptors, such as TCR, BCR, cytokine receptors, and TLRs, can also activate mTOR^41,58,59^. We demonstrated that the CLR family member LSECtin can interact with mTOR and serve as a physiological stimulus of mTORC1. Both in vivo and in vitro studies showed increased phosphorylation of downstream substrate proteins, such as S6K and 4EBP1. Clearly LSECtin, as a member of the CLR family, interacts with mTOR and activates mTORC1, although the exact pathway involved in the LSECtin-mediated mTORC1 activation was not specifically addressed and should be explored in further studies. We believed that these findings provide insights into the roles and mechanism of CLRs in physiological responses.

Billions of cells are cleared on a daily basis in many tissues of the body as part of routine homeostasis^60,61^. mTOR has been reported to be activated upon phagocytosis and/or entosis of cell corpses^61–63^ Our observations showed a similar result that mTORC1 activity was increased after phagocytosis of apoptotic cells in macrophages. When the engulfment was inhibited phagocytosis-induced mTORC1 activation was significantly impaired with reduced p-S6K^T389^ and p-4EBP1^T37/46^. Furthermore, the absence of LSECtin impaired the mTORC1 activity of macrophages induced by apoptotic cells (Fig. 4d), indicating that apoptotic cells could enhance LSECtin-induced mTORC1 activation. An interesting extrapolation from this finding was that apoptotic cells enhance LSECtin-induced mTORC1 activation might by increasing the LSECtin-mTOR interaction, because apoptotic cells could not change the expression of LSECtin (Fig. 4e–h). How does apoptotic cell promote the interaction between LSECtin and mTOR? One possible way that apoptotic cells promoted the interaction between LSECtin and mTOR might be as described below. In the phagocytosis process, a cell deformed its membrane to form a little cone around the apoptotic cell to be absorbed. The engulfed apoptotic cell was thus enclosed within a membrane-bound vacuole called a phagosome. LSECtin was located on the membranes of phagosomes. Phagosomes subsequently fused with lysosomes and formed phagolysosomes. Lysosomes are a scaffolding platform on which mTORC1 becomes activated^64^. Although not specifically addressed, LSECtin and mTOR might meet in this setting; therefore, phagocytic apoptotic cells might enhance the interaction between LSECtin and mTOR. However, this requires further verification, and it would be an interesting question for a future study.

Damage to IECs and the induction of cellular apoptosis are characteristics of IBD^3,65^. Regenerative responses are particularly important in the mammalian gastrointestinal tract, and mucosal healing has emerged as an important end point in clinical trials and as a key goal in IBD therapy^66,67^. During inflammation, additional cells undergo cell death^68^. Clearance of apoptotic cells is associated with reprogramming of phagocytes such as macrophages towards anti-inflammatory states and is part of the process of resolution of inflammation^69^. Intestinal macrophages are considered to be the main participants in establishing and maintaining gut homeostasis^4,70^ and are important scavengers of dead and dying cells^70^. An increasing number of studies have shown that macrophages are novel potential targets for the development of new treatment approaches for IBD^4,6^. Thus, it is believed that understanding how apoptotic cell-engulfing macrophages promote the proliferation of epithelial cells (and thus contribute to tissue homeostasis) during IBD is believed to be critical for developing therapeutic strategies to cure such diseases^9^. LSECtin on macrophages promotes apoptotic cell clearance by macrophages, and induces the production of anti-inflammatory/tissue growth factors in an engulfment-dependent manner, which in turn stimulates IECs proliferation^12^. LSECtin can activate mTORC1. Initial mTORC1 activation in the resting-state may be amplified when the engulfment of apoptotic cells occurs. This later step led to more robust mTORC1 activation, which effectively activated downstream proteins and mediated the function of macrophages. The data presented in this study concluded that phagocytosed apoptotic cells elevate LSECtin-activated mTORC1 signaling and promote the pro-repair function of macrophages after phagocytosis of apoptotic cells. These might partially explain why the more engulfment progress by macrophages through LSECtin is critical for intestinal healing.

Rapamycin is a bacterial macrolide with antifungal and immunosuppressant activities that affects the cell cycle, growth and autophagy, as well as protein synthesis, through suppression of mTORC1 activity^24,71,72^. Rapamycin has potent immunosuppressive properties reflecting its ability to disrupt cytokine signaling that promotes lymphocyte growth and differentiation^73^. Many studies implied that rapamycin prevents effector T cell differentiation and promotes adaptive regulatory T cell lineage commitment^74^. For example, rapamycin exhibits a high neuroprotective activity by suppressing nuclear factor-κB activity^75^; rapamycin can improve the quality of life of patients with multiple sclerosis by decreasing interferon-γ^76^; rapamycin attenuates mouse food allergy through inhibition of the intestinal IL-9 production-mast cell survival axis^77^; rapamycin increase the expression and release of IL-6 (ref. ^78^). Interestingly, the increased usage of rapamycin has been accompanied by inhibited intestinal proliferation. Some studies have found that rapamycin (mTORC1 activation is inhibited) can inhibit intestinal regeneration and lead to impaired intestinal epithelium repair^38,55,79^. Therefore, mTORC1 has a pivotal part in increasing the maintenance of intestinal stem cell activity and proliferation of intestinal epithelium. In LSECtin ΔMφ mice, although intraperitoneal injection of rapamycin systematically inhibited mTORC1 activity, LSECtin expression was restricted to myeloid cells. Similar effects on body weight loss were observed in both LSECtin ΔMφ mice and LSECtin-WT mice when mTORC1 activity was inhibited during DSS-induced colitis, suggesting that LSECtin and mTOR may be involved in the same pathway, and that LSECtin participates in macrophage engulfment of apoptotic cells and regulates intestinal regeneration by activating the mTORC1 pathway. The results of our study provide insight into the mechanisms through which CLRs sense cell death and restore tissue homeostasis. However, how these macrophages promote IECs proliferation in vivo warrants further investigation.

In summary, we demonstrated that the cytosolic N-terminal region of LSECtin interacts with mTOR to enhance mTORC1 activity. Apoptotic cells increased the LSECtin-mTOR interaction and LSECtin-activated mTORC1 in macrophages. Apoptotic cell-mediated activation of mTORC1 in macrophages facilitated the proliferation of IECs and ameliorated colitis. Our study expands our knowledge of the function of CLRs in sensing cell death and restoring tissue homeostasis. Intestinal macrophages are critical for clearing apoptotic epithelial cells and apoptotic neutrophils, reflecting their role as main contributors to the establishment and maintenance of gut homeostasis. Understanding the molecular mechanisms involved in the specification of intestinal macrophages might lead to a new class of targets that promote remission in patients with IBD.

## Materials and methods

### Primary cell isolation

KCs and LPMCs were isolated from colonic tissue using previously described methods^12,80^.

### Animal care and use

Specific pathogen-free (SPF) LSECtin-WT and LSECtin-KO male mice, macrophage-specific LSECtin-KO male mice (Lyz2^-Cre^ LSECtin^fl/fl^, LSECtin ΔMφ) and LSECtin-WT (LSECtin^fl/fl^) mice, and *Lgr5-GFP* male mice with a C57BL/6 genetic background were maintained in individual cages and used when between 6 and 12 weeks of age. As control animals, cohoused and Cre-negative littermate mice were used in all experiments. Animals were randomly assigned to experimental and control groups. All animals were handled in strict accordance with the “Guide for the Care and Use of Laboratory Animals” and the “Principles for the Utilization and Care of Vertebrate Animals”, and all animal work was approved by the Institutional Animal Care and Use Committee (IACUC) at the Beijing Institute of Lifeomics.

### Statistical analysis

Statistical analysis was performed with GraphPad Prism 6 software. Data are presented as the means ± SEM. The statistical data were based on at least three biological replicates. Statistical significance was calculated by unpaired, two-tailed, Student’s *t*-test or one-way analysis of variance where appropriate. A *P* value <0.05 was considered statistically significant. In addition, *P* < 0.05, *P* < 0.01, and *P* < 0.001 are marked with *, **, ***, respectively, in the figures.

## Supplementary information

SUPPLEMENTAL MATERIAL

SUPPLEMENTAL MATERIAL-Figure. S1

SUPPLEMENTAL MATERIAL-Figure. S2

SUPPLEMENTAL MATERIAL-Figure. S3

SUPPLEMENTAL MATERIAL-Figure. S4
