# Supplementary material for: Activation of mTORC1 by LSECtin in macrophages directs intestinal repair in inflammatory bowel disease
Source: Cell Death Dis. 2020 Oct 26;11(10):918. doi: 10.1038/s41419-020-03114-4 (PMC7589503; doi:10.1038/s41419-020-03114-4)
Supplement: Supplementary file 1 — SUPPLEMENTAL MATERIAL [file 41419_2020_3114_MOESM1_ESM.docx]

Activation of mTORC1 by LSECtin in macrophages directs intestinal repair in inflammatory bowel disease

**Supplementary information**

**Materials and methods**

**Cell culture and treatments**

293T cells and RAW264.7 cells were cultivated in Dulbecco’s modified eagle medium (DMEM, Gibco, #11-965-118) supplemented with 10% fetal bovine serum (FBS) (Gibco, #10099-141) and 1% penicillin/streptomycin at 37℃ with 5% CO_2_. Cultured human monocytic THP-1 cells were maintained in Roswell Park Memorial Institute medium (RPMI 1640, Gibco, #C11875500BT) containing 10% FBS and supplemented with 1% penicillin/streptomycin at 37℃ with 5% CO_2_. THP-1 monocytes were differentiated into macrophages after 24 h of incubation with 100 nM phorbol 12-myristate 13-acetate (PMA, Sigma, #P8139) in RPMI medium with 10% FBS and 1% penicillin/streptomycin at 37℃ with 5% CO_2_. HCT116 cells were grown in McCoy's 5A medium (Gibco, #16600-082) supplemented with 10% FBS and 1% penicillin/streptomycin at 37℃ with 5% CO_2_. All cells were monitored by mycoplasma PCR testing and maintained in mycoplasma-free conditions. Cells were transfected with LSECtin and FLAG-tag, HA-tag, and MYC-tag vectors using Lipofectamine 2000 (Invitrogen, #11668) according to the manufacturer's instructions.

**Plasmid and small interfering RNAs (siRNAs)**

Full-length LSECtin and truncated mutants were cloned into the S-SBP-FLAG-tagged vector (pIRES2-EGFP), HA-tagged vector (pCMV-HA), or MYC-tagged vector (pcDNA3.1-MYC). All mutants were generated by site-directed mutagenesis and confirmed by sequencing.

siRNAs were synthesized by JTSBIO Co. (Wuhan, China). For siRNA transfection, cells were transfected twice in 24 h intervals with the indicated siRNA using Lipofectamine RNAiMAX (Invitrogen, #13778) according to the manufacturer’s instructions. The target sequence of the siRNA against human LSECtin was GCGCGAGAACUGUGUCAUGdTdT, and the corresponding sequence in the negative control siRNA was UUCUCCGAACGUGTCACGUdTdT.

**Cell lysis, immunoprecipitation and immunoblotting**

For cell lysis and co-IP, cells were homogenized in RIPA buffer supplemented with protease inhibitor cocktail (Millipore, #539143) and PMSF (20 mM) on ice for 15 min and then sonicated for 15 sec. The lysates were cleared by centrifugation at 12 000 rpm for 12 min at 4°C. Supernatants were collected for immunoprecipitation or immunoblotting.

For anti-FLAG or anti-HA co-IP, anti-FLAG-M2 affinity gel (Sigma, #A2220) or anti-HA affinity gel (Sigma, #E6779) was washed three times with RIPA buffer containing protease inhibitor cocktail. Then, 20 μl of a 50% affinity gel slurry was added to the cleared cell lysates, followed by incubation with rotation for 8 h at 4°C. The beads were washed three times with RIPA buffer containing protease inhibitor cocktail. The immunoprecipitated proteins were denatured by the addition of 60 μl of sample buffer and boiling for 12 min as previously described.

For the reciprocal co-IP, the cells were cultured and processed as described above. Five milligrams of total protein and 5 μg of antibody were first incubated at 4°C for 1 h, and the entire mixture was added to 15 μl of protein G agarose (Sigma, #P7700) and rotated at 4°C for 8 h. The immunoprecipitants were washed 4 times and analyzed for immunoblotting.

For immunoblotting, according to standard protocols, the following antibodies were used: mTOR (Cell Signaling Technology, #2983, 1:1000), phospho-mTOR (Ser2448) (Cell Signaling Technology, #5536, 1:1000), p70 S6 kinase (Cell Signaling Technology, #2708, 1:1000), phospho-p70 S6 kinase (Thr389) (Cell Signaling Technology, #9234, 1:1000), 4EBP1 (Cell Signaling Technology, #9644, 1:1000), phospho-4EBP1 (Thr37/46) (Cell Signaling Technology, #2855, 1:1000), human LSECtin [EPR13724] (Abcam, #ab181196, 1:1000), Rabbit anti-mouse LSECtin polyclonal antibody were produced by our lab (1:1500), FLAG (Sigma, #F3165, 1:3000), HA (BioLegend, #901501, 1:2000), PCNA (Cell Signaling Technology, #13110, 1:1000), cyclin D1 (Life Technologies, #333500, 1:1000), GAPDH (Proteintech, #60004-1, 1:2000), β-actin (Proteintech, #60008-1, 1:2000), and β-tubulin (Proteintech, #66240-1, 1:2000).

**GST-Pull down assay**

For hLSECtin GST-pull down assay, GST and GST-hLSECtin were purified from BL21 *E. coli*, and immobilized onto Glutathione Sepharose 4B at 4°C overnight. 293T cells were transfected with FLAG-mTOR constructs. Then, cells were lysed in the RIPA buffer with the protease inhibitor and incubated with Anti-Flag M2 Affinity beads for 3 h at 4℃. The immunocomplexes were washed three times with RIPA buffer and eluted with TBS buffer (10 mM Tris HCl, 150 mM NaCl, pH 7.4) containing 100 μg/ml FLAG Peptide (Sigma). The eluates were incubated with the Sepharose immobilized with indicated proteins at 4°C for 8 h. Sepharose were then washed three times with the TBS buffer and boiled in 2x SDS loading buffer. Samples were subjected to immunoblot with indicated antibodies and visualized by Coomassie Blue staining.

**LC-MS/MS protein identification**

Cell lysis and co-IP were performed as described above. The coimmunoprecipitated proteins were resolved by 10% SDS-PAGE, stained using Coomassie blue and destained with 20% ethanol and 10% acetic acid.

For sample protein digestion, the gel lanes were cut into cubes (approximately 1×1×1 mm), and these gel cubes were washed twice with 50 mM ammonium bicarbonate. The gel cubes were subsequently subjected to in-gel digestion for 12 h with 15 ng/L sequencing-grade trypsin (Promega, #V511A) at 37℃. Peptides were sequentially extracted by 50% acetonitrile and 0.1% trifluoroacetic acid, 80% acetonitrile and 0.1% trifluoroacetic acid at 37℃ for 30 min. The peptides were desalted using C18 ZipTips (Millipore, #Z720046) and evaporated on a vacuum centrifuge until dry.

For the nano-HPLC MS/MS analysis, nano-LC-ESI-MS/MS was performed on an Orbitrap Fusion Lumos mass spectrometer (Thermo Fisher Scientific) equipped with a U3000 nano-LC system with three replicates. The mobile phases consisted of 0.1% formic acid (solvent A) and 0.1% formic acid in 80% ACN (solvent B). The peptides were loaded onto a C18 trap column (PepMap, Thermo Fisher Scientific, #164946) and separated by a 25 cm column (PepMap, Thermo Fisher Scientific, #164941) at a flow rate of 300 nL/min with a solvent B gradient from 8 to 32% over 60 min. The spray voltage was 2.2 kV in positive mode, and the full scan spectra from m/z 350 to 1800 was generated at a resolution of 60 000. The cycle time was 3 sec, and data-dependent MS/MS spectra were acquired in the orbitrap with a normalized collision energy of 32%. Dynamic exclusion of the fragmented precursor ions was used with the following parameters: an exclusion time of 45 sec and a mass tolerance width of 10 ppm. Single and seven charged species were excluded.

For protein identification and label-free quantification, the acquired mass data were searched against the UniProtKB human database (UniProtKB 2019.02.01) using Proteome Discoverer software (version 2.3, Thermo Fisher Scientific), and the Sequest HT search engine was used. The search parameters were set as follows: enzyme, trypsin; precursor ion mass tolerance, 10 ppm; fragment ion mass tolerance, 0.02 Da; maximum missed cleavages allowed, 2; carbamidomethyl of cysteine residues for static modification; and oxidation of methionine and acetyl of the N-terminal region for dynamic modification. The protein FDR was less than 1.0% and at least one peptide was identified. Label-free quantification analyses were performed on the co-IP assay data of the control group and the LSECtin-overexpressing group or mTOR-overexpressing group.

**Phagocytosis assay**

For co-IP analysis, 1.0×10^7^ hLSECtin-expressing THP-1 cells (after 24 h of incubation with 100 nM PMA) and 6.0×10^6^ mLSECtin-expressing RAW264.7 cells (one day before the phagocytosis assay) were plated in 10 cm culture dishes. Next, the cells were starved for 30 min with medium containing 2% FBS and then incubated with apoptotic HCT116 cells or thymocytes in uptake buffer (medium containing 2% FBS and 0.2% penicillin-streptomycin). Apoptotic cells were used at a 6:1 apoptotic cell/macrophage ratio unless otherwise specified. After incubation for 30 min, the cells were extensively washed with cold PBS and cultured with medium containing 10% FBS for 30 min. Cells were lysed as described above. The protein concentration in the cell fraction lysates was determined using a BCA protein concentration kit (TIANGEN, #PA115) according to the manufacturer’s instructions. The total protein concentration in each group was normalized to perform co-IP as described above.

For the engulfment assay, apoptotic cell-mediated mTORC1 activation with 2.0×10^6^ mLSECtin-expressing Raw264.7 cells, 3.0×10^6^ PMA-stimulated hLSECtin-expressing THP-1 cells, 3×10^6^ KCs, 3×10^6^ BMDMs in a 6-well plate was induced following the protocol previously described. To inhibit engulfment, 1 μM CytoD (Abcam, #ab143484) was added 30 min before the apoptotic thymocytes were added to the cells. Engulfment of apoptotic cells was performed as described above. After the apoptotic cells were washed and cultured with medium containing 10% FBS for 30 min, the cells were lysed, and then, immunoblotting was performed.

**Quantitative RT-PCR (qRT-PCR)**

Following apoptotic cell phagocytosis, BMDMs and KCs were cultured with medium containing 10% FBS for 4 h with or without 1 μM rapamycin. Then, total RNA was extracted using TRIzol (Life Technologies, #15596018) following the manufacturer’s protocol. Typically, 1 μg of total RNA was reverse transcribed into cDNA using a reverse transcription kit (Takara, #RR047A). An applied biosystems ViiA 7 sequence detection system (ABI ViiA 7SDS; Foster City, CA, USA) was used to perform qRT-PCR according to the manufacturer’s protocols. The expression of human *CLEC4G* was normalized to the expression of the housekeeping control gene *ACTIN*. The *ACTIN* forward primer: ATCACCATTGGCAATGAGCG and reverse primer: TTGAAGGTAGTTTCGTGGAT. Human *CLEC4G* primer was purchased from QIAGEN (QIAGEN, #118966293). The expression of mouse target genes was normalized to the expression of the housekeeping control gene *Gapdh*. The following primers were used for the qRT-PCR: *Gapdh* forward primer: AGGTCGGTGTGAACGGATTTG and reverse primer: TGTAGACCATGTAGTTGAGGTCA; *Il10* forward primer: GCTCTTACTGACTGGCATGAG and reverse primer: CGCAGCTCTAGGAGCATGTG; *Hbegf* forward primer: ACCAGTGGAGAATCCCCTATAC and reverse primer: GCCAAGACTGTAGTGTGGTCA; mouse *Clec4g* forward primer: GGTGCCCATCTGGTGATTGT and reverse primer: CAGTGGCTGAAGTTGAGTGAGG.

**Immunofluorescence staining**

Frozen sections of mouse colon tissue were prepared by previously described methods. The tissue sections were incubated in blocking buffer (5% goat serum and 0.3% Triton-X 100 in PBS) at room temperature for 1 h followed by staining with primary antibodies for 16 h at 4℃. The following primary antibodies were used: anti-pan cytokeratin antibody (Abcam, #ab7753, 1:500) and anti-GFP antibody (Abcam, #ab183734, 1:300). Then, the slides were washed and incubated for 1 h with the following secondary antibodies: goat anti-mouse TRITC and goat anti-rabbit Alexa Fluor-488 (Jackson ImmunoResearch Laboratories, 1:200). The sections were counterstained with 4’,6-diamidino-2-phenylindole dihydrochloride (DAPI) before being mounted.

For cellular immunofluorescence, cells were grown on coverslips and after phagocytosis assays (apoptotic thymocytes were stained by Hoechst (Thermo Scientific, #62249, 2 μM) before phagocytosis assays). Cells were fixed with 4% paraformaldehyde for 10 min at room temperature, washed with PBS for 3 times. Cells were incubated in 50 mM NH_4_Cl in PBS for 10 min. Next, cells were incubated in binding buffer (0.1% Saponin, 0.2% BSA in PBS) for 30 min and then incubated with primary antibodies anti-mTOR (Cell Signaling Technology, #2983, 1:200) and anti-HA (BioLegend, #901501, 1:100) at 4 °C overnight. Then incubated secondary antibodies: goat anti-mouse Alexa Fluor-488 and goat anti-rabbit TRITC (Jackson ImmunoResearch Laboratories, 1:200).

All immunofluorescence staining was performed in the dark. Imaging was performed using a Zeiss LSM 880 microscope, and images were processed using Zeiss ZEN software.

**DSS-induced colitis model**

Littermates from LSECtin^+/-^/Lyz2^-Cre^ LSECtin^fl/-^ crossbred parents were used for the experiments. To induce of colitis, male mice received 2-2.5% (wt/vol) DSS (wt. 36 to 50 kDa, MP Biomedicals, #160110) in their drinking water for 5 days, followed by 4 days of untreated water. The DSS water was replaced on day 3. On day 9, the mice were sacrificed and assessed for colitis.

For the rapamycin treatment, mice were administered an intraperitoneal injection of rapamycin at 10 mg/kg/day or vehicle every day while ingesting DSS. Mice received 2% (wt/vol) DSS in their drinking water for 7 days, followed by untreated water. The colon lengths were straightened, but not stretched, and measured.

All animals were randomly assigned to experimental and control groups by one person, and then another person randomly and blindly monitored the weights of all mice daily.

**Supplementary Figure Legends**

**Fig. S1 Identification of mTOR as an LSECtin-interacting protein. a** Analysis of FLAG-hLSECtin overexpression (left panel) and FLAG co-IP (right panel). **b** Samples of the FLAG-hLSECtin or FLAG coimmunoprecipitated proteome subjected to SDS-PAGE before LC-MS/MS. **c** Venn diagrams show the overlap of nonredundant quantified proteins captured by hLSECtin in three replicates. **d** Pearson correlation coefficient showing the pairwise correlation of protein intensity between replicate experiments. The Pearson correlation coefﬁcient was indicated. **e** THP-1 cells were transfected with the indicated siRNA, and 48 h later, hLSECtin expression was determined. **f** Analysis of FLAG-mTOR overexpression (left panel) and FLAG co-IP (right panel). Samples of the FLAG-hLSECtin or FLAG coimmunoprecipitated proteome were subjected to SDS-PAGE before LC-MS/MS. All experiments were repeated three times.

**Fig. S2 Validation of mTOR as an LSECtin-interacting protein. a** Schematic depicting hLSECtin isoforms and truncation mutants. ICD: intracellular cytoplasmic domain, TM: transmembrane domain, ECD: extracellular domain, CRD: carbohydrate recognition domain. **b** Coimmunoprecipitates were prepared from mLSECtin-expressing RAW264.7 cells and analyzed along with cell lysates by immunoblotting for detection of the indicated proteins. All experiments were repeated three times. **c** The localization of mLSECtin and mTOR in HA-mLSECtin-expressing RAW264.7 cells after engulfing apoptotic thymocytes was investigated by immunofluorescence. All experiments were repeated three times.

**Fig. S3 LSECtin facilitates the activation of mTORC1 signaling *in vitro* and *in vivo*. a** Schematic depicting hLSECtin isoforms and truncation mutants. ICD: intracellular cytoplasmic domain, TM: transmembrane domain, ECD: extracellular domain, CRD: carbohydrate recognition domain. **b**, **c** The distribution of hLSECtin (**b**) and their intracellular fragments (**c**) in 293T cells and THP-1 cells. **d** Schematic depicting mLSECtin isoforms and truncation mutants. **e** PCR detection of LSECtin in the genomic DNA of the LSECtin-WT and LSECtin-KO mice based on samples taken from mouse tails (left panel). qRT-PCR detection of mLSECtin in LSECtin-WT and LSECtin-KO mice in liver KCs (right panel); all genes were presented relative to the expression of *Gapdh*. All experiments were repeated three times. Data were calculated by unpaired, two-tailed Student’s t-test; ***P< 0.001.

**Fig. S4 Elevated mTORC1 signaling promotes the pro-repair function of macrophages after phagocytosis of apoptotic cells. a** Schematic of flow cytometry analysis of BMDMs. More than 90% of the adherent cells were CD11b and F4/80 double positive. **b** Schematic for DSS-induced colitis in mice with or without rapamycin treatment.
