# Supplementary figures and images for: Activation of mTORC1 by LSECtin in macrophages directs intestinal repair in inflammatory bowel disease
Source: Cell Death Dis. 2020 Oct 26;11(10):918. doi: 10.1038/s41419-020-03114-4 (PMC7589503; doi:10.1038/s41419-020-03114-4)

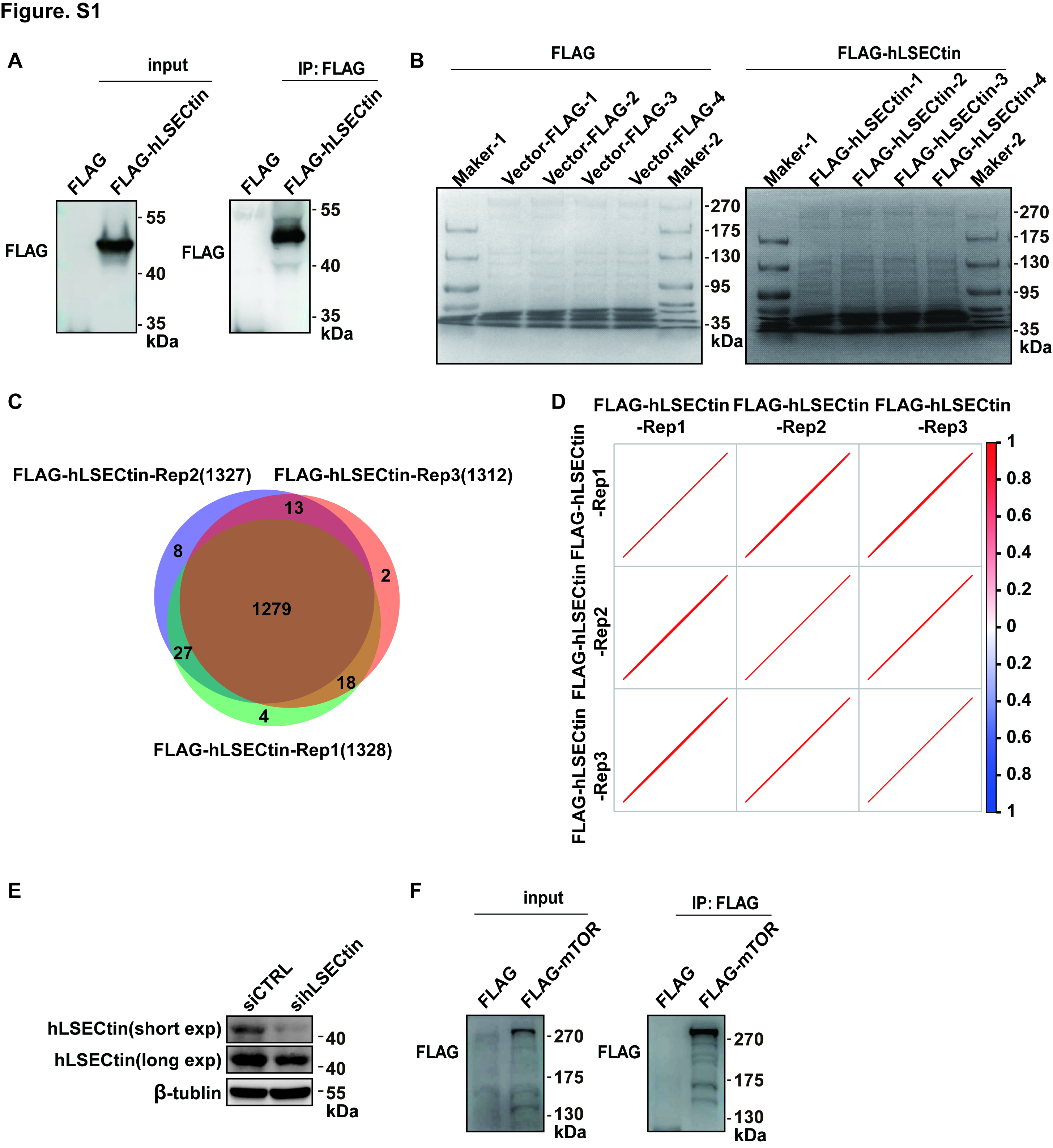

Supplement: Supplementary file 2 — SUPPLEMENTAL MATERIAL-Figure. S1 [file 41419_2020_3114_MOESM2_ESM.tif]

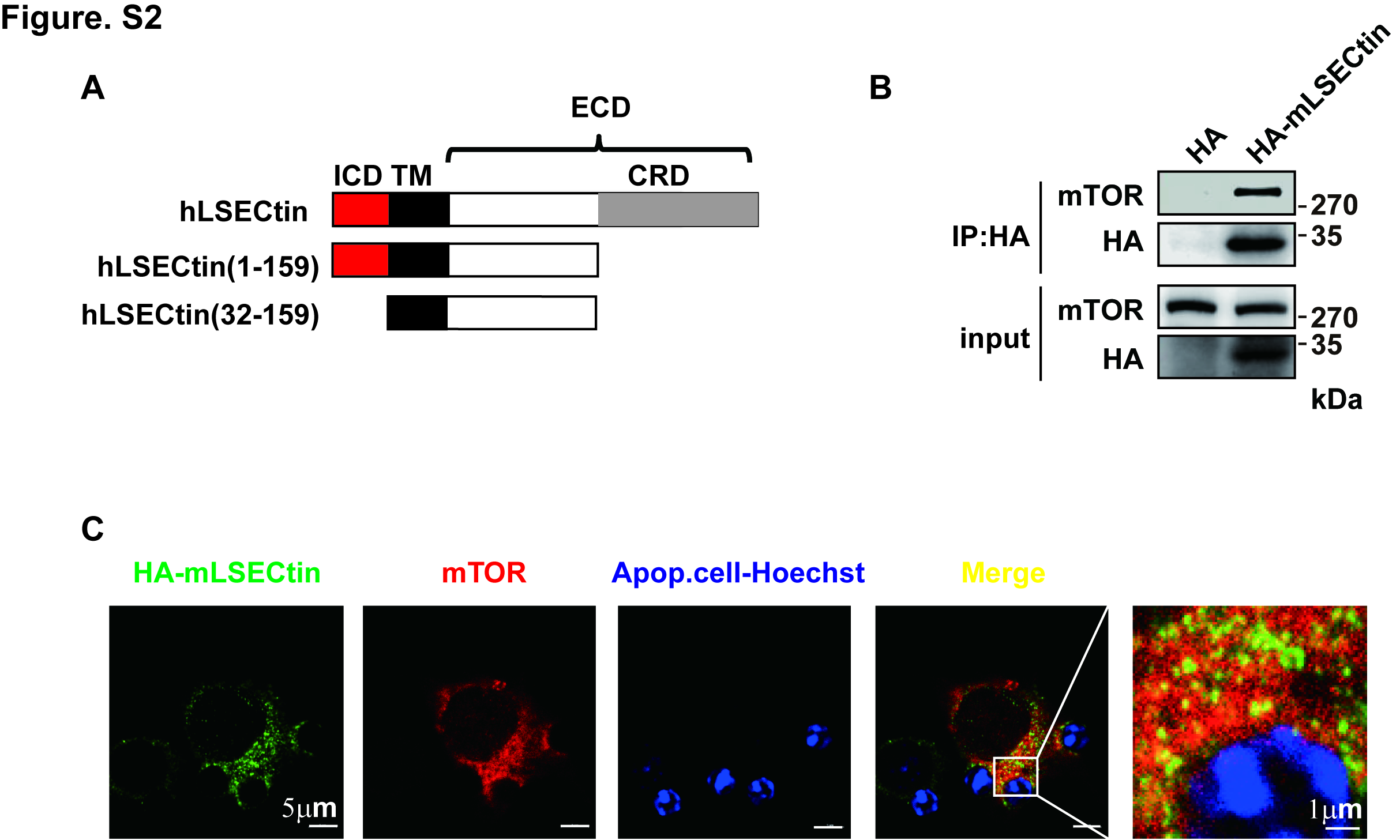

Supplement: Supplementary file 3 — SUPPLEMENTAL MATERIAL-Figure. S2 [file 41419_2020_3114_MOESM3_ESM.tif]

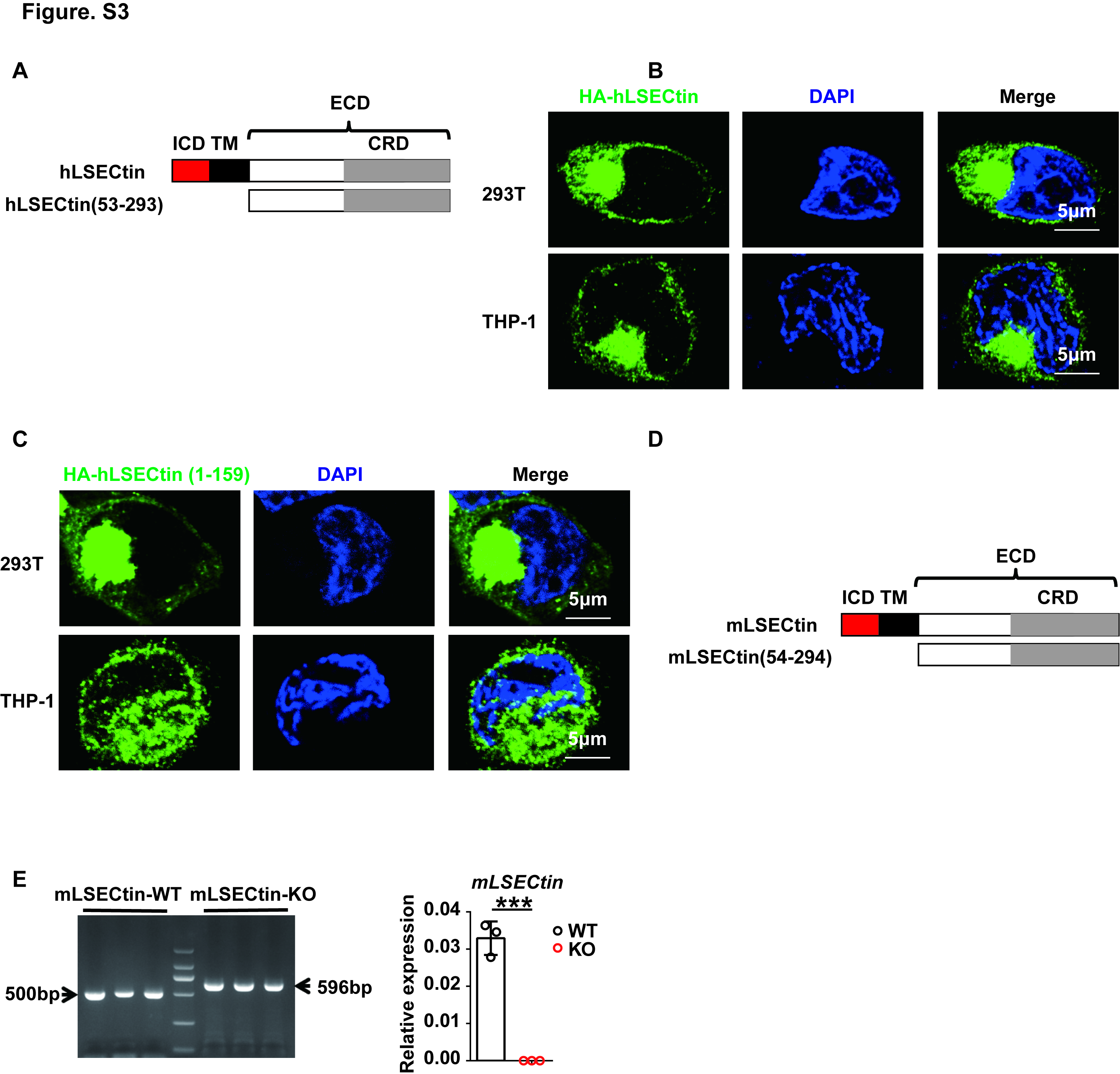

Supplement: Supplementary file 4 — SUPPLEMENTAL MATERIAL-Figure. S3 [file 41419_2020_3114_MOESM4_ESM.tif]

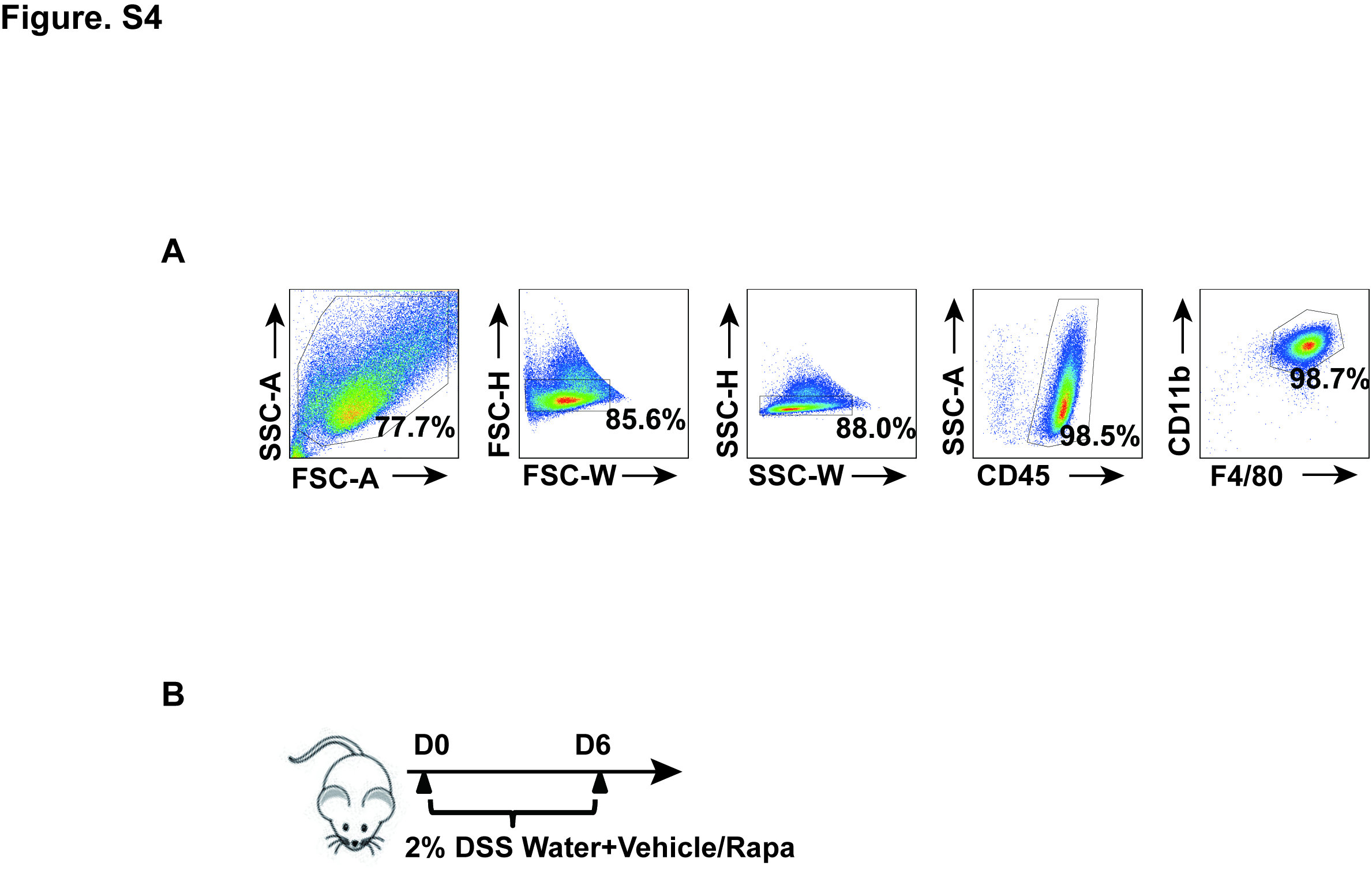

Supplement: Supplementary file 5 — SUPPLEMENTAL MATERIAL-Figure. S4 [file 41419_2020_3114_MOESM5_ESM.tif]
